# Small but Powerful: Top Predator Local Extinction Affects Ecosystem Structure and Function in an Intermittent Stream

**DOI:** 10.1371/journal.pone.0117630

**Published:** 2015-02-25

**Authors:** Pablo Rodríguez-Lozano, Iraima Verkaik, Maria Rieradevall, Narcís Prat

**Affiliations:** Freshwater Ecology and Management (F.E.M.) Research Group, Departament d’Ecologia, Facultat de Biologia, Universitat de Barcelona, Barcelona, Spain; Dauphin Island Sea Lab, UNITED STATES

## Abstract

Top predator loss is a major global problem, with a current trend in biodiversity loss towards high trophic levels that modifies most ecosystems worldwide. Most research in this area is focused on large-bodied predators, despite the high extinction risk of small-bodied freshwater fish that often act as apex consumers. Consequently, it remains unknown if intermittent streams are affected by the consequences of top-predators’ extirpations. The aim of our research was to determine how this global problem affects intermittent streams and, in particular, if the loss of a small-bodied top predator (1) leads to a ‘mesopredator release’, affects primary consumers and changes whole community structures, and (2) triggers a cascade effect modifying the ecosystem function. To address these questions, we studied the top-down effects of a small endangered fish species, *Barbus meridionalis* (the Mediterranean barbel), conducting an enclosure/exclosure mesocosm experiment in an intermittent stream where *B. meridionalis* became locally extinct following a wildfire. We found that top predator absence led to ‘mesopredator release’, and also to ‘prey release’ despite intraguild predation, which contrasts with traditional food web theory. In addition, *B. meridionalis* extirpation changed whole macroinvertebrate community composition and increased total macroinvertebrate density. Regarding ecosystem function, periphyton primary production decreased in apex consumer absence. In this study, the apex consumer was functionally irreplaceable; its local extinction led to the loss of an important functional role that resulted in major changes to the ecosystem’s structure and function. This study evidences that intermittent streams can be affected by the consequences of apex consumers’ extinctions, and that the loss of small-bodied top predators can lead to large ecosystem changes. We recommend the reintroduction of small-bodied apex consumers to systems where they have been extirpated, to restore ecosystem structure and function.

## Introduction

Predation is an important species interaction that has implications for biological populations, communities, and ecosystems. In addition to affecting prey abundance and distribution, predation affects other non-prey taxa and ecosystem processes through indirect pathways [1,2]. In recent decades, human activity has caused the extinction of many apex consumers (i.e., top predators) [3,4], and several studies have indicated subsequent ecosystem changes that are complex, unpredictable, and largely unknown [4,5]. Given that current biodiversity loss is biased towards species in the higher trophic levels [3,6], the ecosystem impacts of top-predator decline remain a research priority [7].

The extinction of top predators is often associated with an increase in mesopredators [8–10], i.e., any mid-ranking predator in a food web. An ecosystem may have several mesopredators, and a mesopredator in one system may be a top predator in another system [8]. ‘Mesopredator release’ often leads to a decrease in the prey [9,10], a straightforward conclusion, termed a ‘trophic cascade’, when each trophic level is connected in a direct and negative way [9,11,12]. But, as showed in a recent review about apex-mesopredator-prey interactions [10], not all trophic webs have a linear shape. From the 32 studies, Brashares *et al*. [10] found that 40% of the interactions were triangular: those in which top predators feed on mesopredators and also on prey, resulting in intraguild predation (IGP; characterised by predators that feed on other predators with which they share prey taxa). If IGP occurs, the apex consumer exerts top-down control on both mesopredator and prey, and then, apex consumer extinction would liberate mesopredator and prey from its top-down structuring forces. However, in that case, ‘mesopredator release’ could also lead to an increase on prey top-down control, neutralising apex consumer loss. This would result in a negative or a null net effect on prey taxa, and consequently, dampen the trophic cascade on primary production [13–15]. In addition, according to the predator-mediated coexistence theory [16] and to recent modelling work [17], apex consumer loss can cause secondary extinctions in adjacent and non-adjacent trophic levels [12,18,19], mainly because predators can facilitate coexistence among prey species. Thus, top predator extinctions have been related not only to an increase in mesopredator abundance but also to a decline in biodiversity [9,12].

Intermittent streams are present in all climate areas and are ecologically unique [20,21], but most research in these systems focused on how hydrological variability shapes community attributes and biogeochemical processes [21,22], while the role of top-down structuring forces has been largely overlooked. Furthermore, intermittent streams often lack large aquatic consumers that are often considered to be top predators, and instead, are typically inhabited by predaceous invertebrates and small-bodied fish [23,24]. These systems have been considered a refuge from vertebrate predation [23,25], and even from invertebrate predation, as some studies suggest predatory invertebrates have lower abundances in intermittent than in permanent streams [26]. Other research evidence indicates that predation pressure increases with stream fragmentation in isolated pools, typically in summer, when predatory lentic invertebrates (odonates, hemipterans and coleopterans) replace reophilous taxa [27–30]. Regarding predatory vertebrates, previous studies of intermittent streams show that predatory fish can affect stream macroinvertebrates in terms of: whole community assemblage and total density [31], the densities of specific groups (e.g., air breathing macroinvertebrates [32]), total biomass [33], and prey body condition [34]. Other studies suggest that predatory fish have no effect on macroinvertebrate communities [35]. All these studies were performed in dry season conditions, in isolated pools or in pools that became isolated during the experiment, when predation pressure reaches its peak in these systems. The importance of predation in intermittent streams during periods of flow remains unknown.

**Fig 1:**
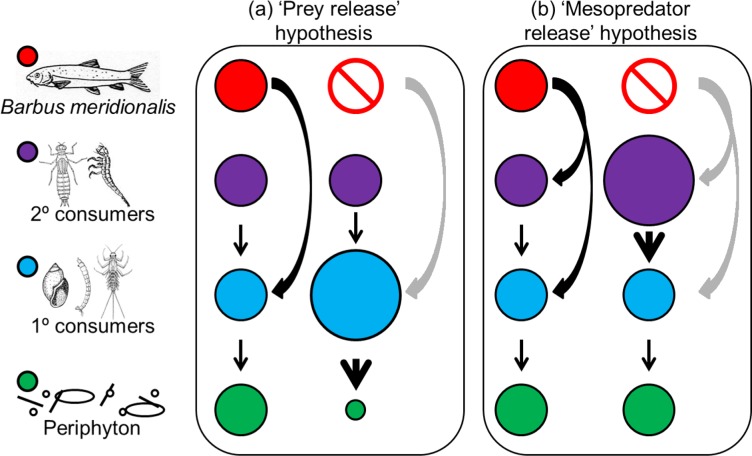
Diagram of the trophic interactions in intermittent stream food webs in the presence and absence of the apex consumer. This diagram describes our two hypotheses related to apex consumer extirpation: a) ‘prey release’ hypothesis and b) ‘mesopredator release’ hypothesis. Circumference size in top predator absence diagrams represents the density decrease, increase or persistence compared to the top predator presence diagram. Arrows represent trophic interactions. Thicker arrows = magnified trophic interactions due to apex consumer extirpation; grey arrows = lost trophic interactions after apex consumer extirpation.

The objective of our research was to determine if the loss of an endangered apex consumer from an intermittent stream would result in major changes to ecosystem structure and function. *Barbus meridionalis* (A. Risso, 1827), also known as the Mediterranean barbel, is an endemic small-bodied fish in the Mediterranean intermittent streams of Spain and France, and often act as apex consumer in these ecosystems. This species is considered ‘vulnerable’ in the Spanish Red Book and ‘near threatened’ internationally. We studied the top-down impacts of *B*. *meridionalis* to determine if the loss of the top predator (1) leads to a ‘mesopredator release’, affecting primary consumers and changing whole community structure, and (2) triggers a cascade effect modifying ecosystem function (i.e., periphyton primary production). *Barbus meridionalis* has been classified as an insectivore benthic species [36] that feeds primarily on chironomid larvae, detritus (which could be explained by its benthic feeding behaviour), mayflies and isopods (mainly primary consumers [37]). Thus, apex consumer extirpation might not lead to ‘mesopredator release’, and instead could promote a trophic cascade resulting in ‘prey release’ and lower primary production (i.e., ‘prey release’ hypothesis, see Fig. 1A). Alternatively, *B*. *meridionalis* could feed on two trophic levels (i.e., macroinvertebrate secondary and primary consumers), in which case top predator removal would trigger a ‘mesopredator release’ due to IGP. According to IGP theory, ‘mesopredator release’ could compensate apex consumer extirpation in terms of prey top-down control, and the trophic cascade would be dampened with no impact on prey or primary production (i.e., ‘mesopredator release’ hypothesis, see Fig. 1B). To address these questions, we performed a field experiment using enclosure/exclosure mesocosms in a Mediterranean stream where *B*. *meridionalis* became locally extinct following a wildfire.

## Methods

### Ethics statements

This study was authorized by the Autonomous Government of Catalonia (Generalitat de Catalunya) and the Natural Parks Department of the Government of Barcelona (Diputació de Barcelona). The University of Barcelona reviewed and approved the project without requirement for ethics approval. Fish were euthanized following the standard protocol recommended by the animal welfare service at the University of Barcelona (anaesthetized using Tricaine methanesulfonate (MS- 222)), and all efforts were made to minimize animal stress and suffering during this study.

### Study area

The Vall d’Horta stream (41°40’24’‘N, 2°02’4’‘E; Altitude: 480 m asl) is a first order stream located in the protected area of Sant Llorenç del Munt i l’Obac Natural Park (50 km inland from Barcelona, NE Spain). The main stream course is formed from the confluence of the Pregona and Font del Llor creeks draining to the Ripoll‘s Basin (a tributary of the Besòs River). This hilly area is characterised by a Mediterranean climate and a calcareous geology, with alternating highly permeable and less permeable substrates where springs are located (see [27,38] for a detailed site description). *Barbus meridionalis* is a common fish within these intermittent streams that find refuge in the remaining permanent pools during periods of hydrological disconnection (usually in summer). In August 2003, a wildfire burned a forested area of 4543 ha, affecting 62% of the Vall d’Horta basin. As a consequence of this wildfire, *B*. *meridionalis* became locally extinct in some of the affected streams, even in the pools, potentially due to chemical changes that occurred during the first rainfall events [39]. The fish population has not recovered since the fire, most likely due to natural and human barriers in the lower part of the study site.

We conducted the experiment in a 100 m reach formed by a large pool where riparian vegetation was not burned by the wildfire. This reach was selected because, as observed in the years before the fire, barbels took refuge in these pools to survive periodic drought conditions present in the area when intermittent Mediterranean streams were reduced to isolated pools [40]. Physicochemical water analyses (n = 9) were performed before, during, and at the end of the experiment. The results (presented as the mean ± SE) confirmed that water of this reference stream was hard (conductivity: 520 ± 5 μS cm^-1^; pH: 7.9 ± 0.1) and oligotrophic (N-NO_3_^-1^: 0.29 ± 0.02 mg l^-1^; N-NH_3_: 0.019 ± 0.003 mg l^-1^; P-PO_4_^3-^<0.01 mg l^-1^). The stream discharge averaged 15.7 ± 0.9 l s^1^, which, with the very low water velocity in the pool (< 1 cm s^-1^), naturally kept the pool water renewed and oxygenated (DO_2_: 9.6 mg l^-1^, 84.7%) during our study.

### Mesocosm design

We performed an enclosure/exclosure mesocosm experiment to manipulate *B*. *meridionalis* densities. Removal experiments that simulate the loss of one or more species from a natural community can reveal the consequences of apex consumer extinctions and assess biodiversity-ecosystem function (BD-EF) relationships [41].

**Fig 2:**
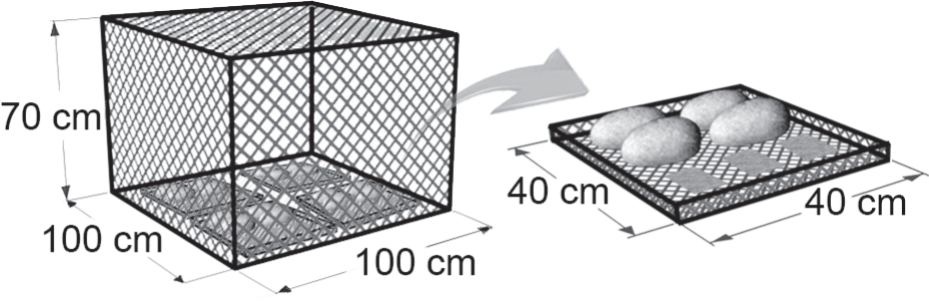
Diagram of the experimental enclosure. Diagram of the experimental enclosure and one of the four identical trays that contained stones for macroinvertebrate colonisation and glass tiles for periphyton colonisation. Dimensions are indicated.

We used nine large cages (100 x 100 cm surface, 70 cm height; see Fig. 2) covered with a 10 mm mesh that retained fish but allowed macroinvertebrate emigration/immigration, thereby minimising the impact of our experimental design on the rate of prey exchange with the benthos [42,43]. In each cage, four plastic trays (40 x 40 cm surface, bottom of 1 mm mesh size) were used as replicates (36 trays in total); each tray contained four medium-sized stones for macroinvertebrate colonisation and three glass tiles (2 x 4 cm) for periphyton colonisation (see Fig. 2). Tray substrates within the mesocosms were complex due to the material deposited during the colonisation period; substrate was formed by a mixture of sediment, detritus and leaves, which provided some refuge to invertebrates [44,45] along with the initial added stones. To study the consequences of *B*. *meridionalis* extirpation, we tested three treatments with varying barbel density levels in the enclosures: i) no fish; ii) barbels at low density (i.e., 2 individuals m^-2^, the known pre-fire density; A. de Sostoa pers. comm.); and iii) barbels at high density (i.e., 4 individuals m^-2^, twofold the pre-fire density). Barbels were caught using an electrofishing source downstream from our study site, and individuals selected for the experiment were approximately the same size (total length 101.8 ± 2.6 mm; mean ± SE) and weight (2.3 ± 0.2 g). To ensure similar initial conditions, barbels were kept in observation for 24 h before starting the experiment after electrofishing and transportation.

### Sampling and laboratory protocols

The field experiment was conducted in late spring of 2010 before pool disconnection (flow averaged 15.7 ± 0.9 l s^1^), over the course of five weeks. Three weeks were allowed for periphyton and macroinvertebrate colonisation, a time previously described as adequate for equilibrating the mesocosm and background macroinvertebrate densities [46]. Two weeks were allowed for barbel interaction. During the colonisation period, the cage tops were opened to facilitate aerial colonisation and the entrance of organic material. Before the addition of barbels to the experimental enclosures, one tray per cage (n = 9) was removed and sampled to test if there were differences in colonisation among cages. Barbel density levels were randomly assigned to enclosures, and the cage tops were closed following barbel introductions to avoid bird or mammal predation. After two weeks of interaction, mesocosms were destructively sampled with the same effort for each tray (n = 27; 9 trays per treatment). Tray contents (with stones) were carefully washed in a 250 μm mesh sieve and preserved in 4% formalin until being processed in the laboratory. All samples were sorted, counted and identified. Taxonomic resolution was primarily to the genus level, including Chironomidae. Some Diptera were identified to the family level, and Oligochaeta, Ostracoda, Cladocera, Copepoda, Hydracarina and terrestrial invertebrates identified to higher levels. Each taxon was categorised as either secondary or primary consumer according to Merritt and Cummins and Tachet *et al*. [47,48]. Periphyton net primary production was measured as the net accumulation of chlorophyll-*a* on artificial substrata [49]. Chlorophyll-*a* was measured after extraction in acetone (90%) for 24 h in the dark at 4°C, sonication for 5 min at 40 kHz, and filtration (GF/F Whatman 0.7 μm-pore size). Following Jeffrey and Humphrey [50], chlorophyll-*a* concentration was determined spectrophotometrically (Perkin-Elmer, Lambda UV/VIS).

In order to test if *B*. *meridionalis* also feeds on predatory invertebrates (not only on primary consumers), and therefore, if intraguild predation occurs, we analysed barbels’ gut contents. Barbels were euthanised using an overdose of anaesthetic (MS-222). Gut contents were preserved in 4% formalin, sorted, counted, and identified at the same taxonomic level as the benthic samples.

### Data analysis

To test differences among the three barbel density treatments, we used the non-parametric Kruskal-Wallis test (K-W test). Then, pairwise Mann-Whitney *U*-tests were used to detect significant differences between treatments. We compared total macroinvertebrate abundance (total number of individuals m^-2^), taxa richness (number of different taxa), rarefied taxa richness (taxa richness corrected by macroinvetebrate abundance in the sample), Simpson’s diversity index (*D*, calculated as $D=\underset{i}{\Sigma }(n_{i}(n_{i}-1)/N(N-1))$, where *n*_*i*_ is the number of individuals of taxon *i* and *N* is the total number of macroinvertebrates [51]), abundance of common taxa (number of individuals of each abundant taxon, i.e., > 50 ind m^-2^ in the treatment lacking barbels), and periphyton net primary production (net accumulation of chlorophyll-*a*) among the three treatments.

We used permutational multivariate analysis of variance (PERMANOVA, ‘Adonis’ function in R) on the Bray-Curtis distance matrix, after the log-transformation of the macroinvertebrate abundance data, to test differences in macroinvertebrate community composition among treatments. Afterwards, we used indicator species analysis, using ‘IndVal’ test in R, to identify which taxa of the macroinvertebrate communities could serve as indicator for each barbel density treatment. The ‘IndVal’ test calculated the indicator value for each taxon, combining measurements of taxon specificity to each established barbel density treatment with taxon fidelity within each treatment [52]. The significance of ‘IndVal’ measures was tested using the Monte Carlo test with 9999 permutations.

We also calculated predator:prey ratios for abundance and richness, dividing the abundance (or richness) of secondary consumers by that of primary consumers for each sample. To test for intraguild predation, we also categorised each taxon found in the gut contents as either primary or secondary consumer, and calculated the proportion (%) of each category in the contents. All statistical analyses were performed in R 2.15.2., we used ‘vegan’ and ‘labdsv’ packages [53].

## Results

We found 81 taxa (76 aquatic invertebrates, 1 amphibian, and 4 terrestrial invertebrates) throughout the mesocosm experiment. Macroinvertebrate communities in the mesocosm were similar to those found during previous research in the stream [38]. Primary consumers were typically chironomids, mayflies (such as *Habroplebia* sp. *Baetis* sp. or *Caenis* sp.), gastropods (such as *Gyraulus* sp. or *Radix* sp.) and crustaceans (Cladocera and Ostracoda); while secondary consumers were dominated by predatory chironomids (*Zavrelimyia* sp. and *Procladius* sp.), water beetles (mainly from Dytiscidae family), hemipterans (*Parasigara* sp.), Odonates (such as *Chalcolestes viridis*, *Sympetrum* sp. or *Aeshna* sp.) and leeches (*Helobdella stagnalis*) (S1 Table). Community-level analyses of the macroinvertebrate samples before the addition of barbels to the enclosures showed a homogeneous colonisation of the experimental cages. Total macroinvertebrate density, taxa richness, Simpson’s diversity index, and community composition did not differ among cages (K-W tests, p>0.1; Adonis, F = 0.69, p = 0.87). Similarly, significant differences in periphyton net primary production were not observed (K-W test, χ^2^ = 0.39, p = 0.83).

**Fig 3:**
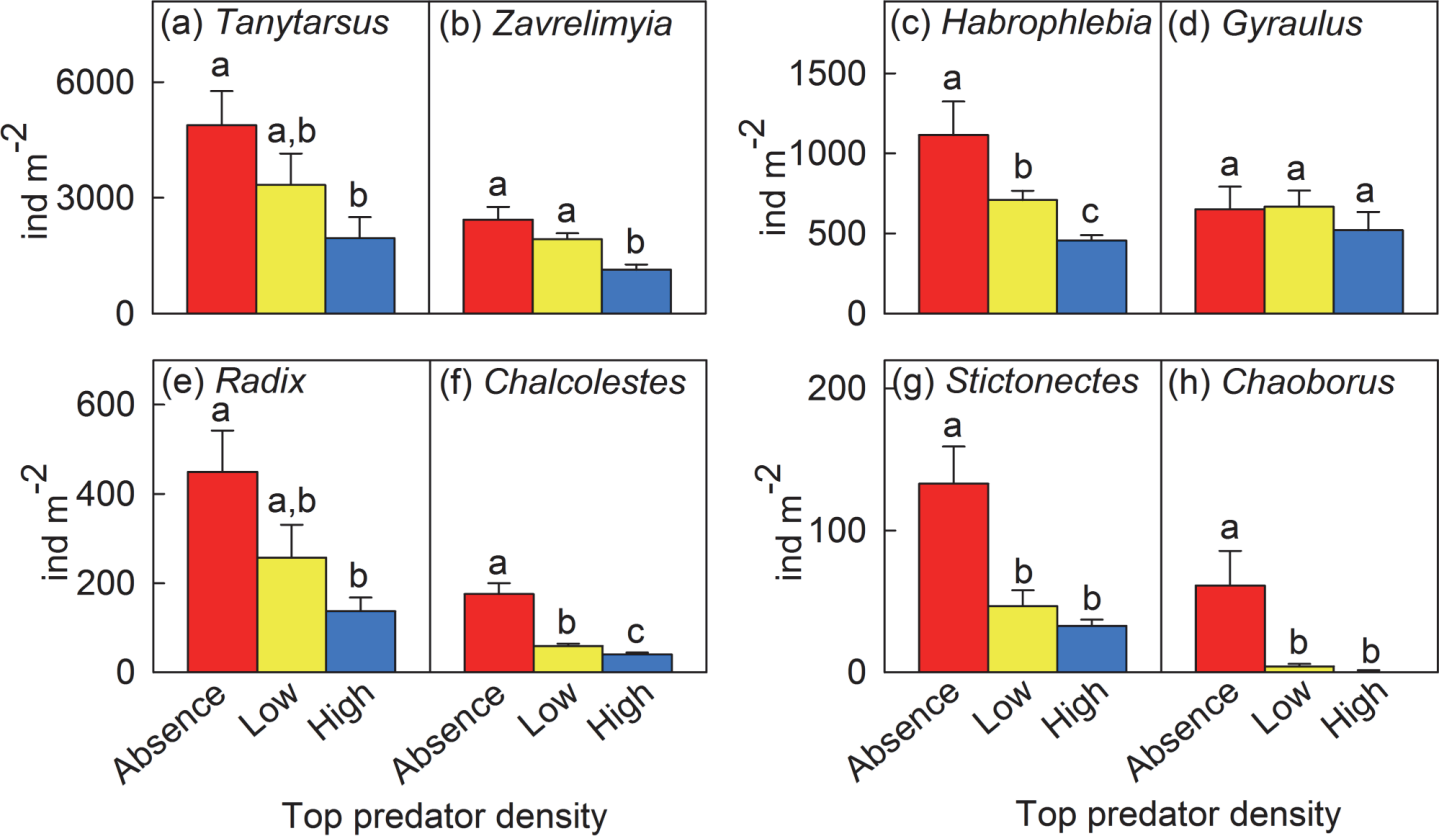
Macroinvertebrate abundance for eight common taxa in the three barbel treatments. Macroinvertebrate abundance for eight of the most abundant taxa (> 50 ind m^-2^ in the treatment lacking barbels) in the three treatments with varying *B*. *meridionalis* densities. Bars represent mean ± SE (individuals m^-2^). Graphs are sorted by taxa abundance: (a) *Tanytarsus* sp., (b) *Zavrelimyia* sp., (c) *Habrophlebia* sp., (d) *Gyraulus* sp., (e) *Radix* sp., (f) *Chalcolestes viridis*, (g) *Stictonectes* sp. and (h) *Chaoborus* sp. Red bars = treatment without barbels; yellow bars = treatment with a low density of barbels; blue bars = treatment with a high density of barbels. Different letters correspond to significant differences resulting from the pairwise comparisons among treatments (*U*-test, p<0.05).

Barbel presence reduced macroinvertebrate total density (χ^2^ = 9.09, p = 0.011); macroinvertebrate density declined almost by half (46.2%) in the treatment with high barbel density compared to the treatment that did not contain barbels (*U* = 12, p = 0.01). We did not detect significant differences among treatments in taxa richness (χ^2^ = 4.29, p = 0.12) or in the Simpson’s diversity index (χ^2^ = 0.77, p = 0.68). The density of the most abundant macroinvertebrate taxa declined when barbels were present, but vulnerability varied among prey (Fig. 3, S1 Table). We distinguished four patterns of abundance related to barbel density: i) a decrease in abundance proportional to barbel density for some taxa such as *Habrophlebia* sp. and *Chalcolestes viridis* (see Fig. 3C,F); ii) a sharp decrease in abundance at barbel presence (i.e., at both low and high barbel densities but not proportional to barbel presence) for other taxa (e.g., mobile predators *Stictonectes* sp. and *Chaoborus* sp.; see Fig. 3G-H); iii) a significant reduction in taxa abundance only at high barbel density treatment compared to the other treatments (e.g. *Zavrelimyia* sp.; see Fig. 3B); and iv) no change in abundance for other taxa irrespective of barbel densities (e.g., *Gyraulus* sp.; see Fig. 3D).

There were significant differences among the three treatments in the composition of macroinvertebrate communities (Adonis, F = 2.39, p<0.001). Twelve taxa were identified as indicators in the treatment that did not contain barbels (Table 1) and two taxa in the low barbel density treatment. No indicator taxa were found in the high barbel density treatment.

**Table 1:** Macroinvertebrate taxa detected as significant indicators for the three barbel density treatments.

Taxa	T	IndVal	P
*Chaoborus* sp.	1	72.05	<0.001
*Cloeon* sp.	1	70.88	<0.001
*Parasigara* sp.	1	69.02	<0.001
*Procladius* sp.	1	65.10	0.008
Chalcolestes viridis	1	64.04	<0.001
*Agabus* sp.	1	63.40	0.010
*Stictonectes* sp.	1	62.69	<0.001
Ostracoda	1	56.53	0.002
Cladocera	1	55.97	0.010
*Radix* sp.	1	53.33	0.019
*Habrophlebia* sp.	1	48.90	<0.001
*Zavrelimyia* sp.	1	44.30	0.012
*Oulimnius* sp.	2	56.56	0.007
Copepoda	2	49.97	0.021

T—Treatments: 1 = treatment without barbels, 2 = treatment with a low density of barbels. IndVal—indicator value. P—its respective *p*-value.

**Fig 4:**
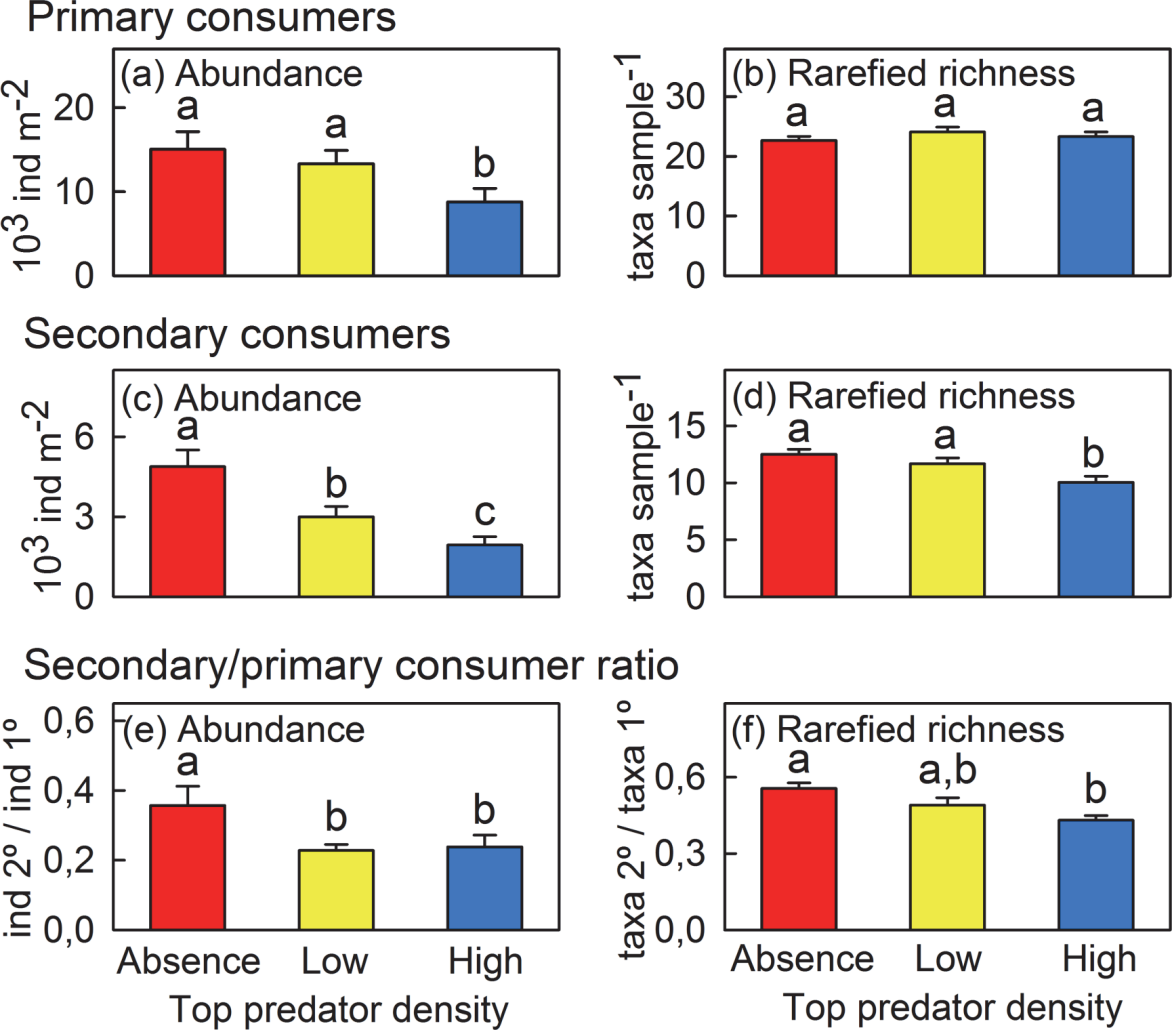
*Barbus meridionalis* density effects on macroinvertebrate abundance and rarefied richness for primary and secondary consumers. *Barbus meridionalis* density effects on macroinvertebrate abundance (mean ± SE individuals m^-2^) and rarefied taxa richness (mean ± SE rarefied taxa sample^-1^) for: (a-b) primary consumers, (c-d) secondary consumers, and (e-f) the ratio of secondary to primary consumers (mean ± SE ratio sample^-1^). Red bars = treatment without barbels; yellow bars = treatment with a low density of barbels; blue bars = treatment with a high density of barbels. Different letters correspond to significant differences resulting from the pairwise comparisons among treatments (*U*-test, p<0.05).

When we analysed macroinvertebrate communities separately for primary and secondary consumers, we detected that *B*. *meridionalis* density affected primary consumer abundance (χ^2^ = 7.38, p = 0.025; Fig. 4A) but not primary consumer richness (χ^2^ = 1.19, p = 0.55) or rarefied richness (χ^2^ = 1.42, p = 0.49; Fig. 4B). Top predator absence increased secondary consumer abundance (χ^2^ = 12.49, p = 0.002; Fig. 4C) and richness before (χ^2^ = 12.89, p = 0.002) and after rarefaction (χ^2^ = 8.17, p = 0.017; Fig. 4D). The ratio for predator:prey abundance marginally increased (abundance: χ^2^ = 5.40, p = 0.07, Fig. 4E) in the absence of barbels, whereas the ratio for predator:prey richness increased significantly (richness: χ^2^ = 12.00, p = 0.002; rarefied richness: χ^2^ = 9.92, p = 0.007; Fig. 4F).

Gut content analysis revealed that predatory invertebrates (secondary consumers) amounted to, on average, 22.8 ± 3.5% (mean ± SE) of the individuals in the barbels’ gut contents. The most abundant predators found in the gut contents were *Zavrelimyia* sp. (a chironomid), *Parasigara* sp. (an hemipteran), and *Stictonectes* sp. (a water beetle). Other predatory invertebrates including Odonates (such as Lestidae, Libellulidae and Aesnidae families) and other water beetles (such as *Agagus* sp. or *Nebrioporus* sp.) were also found in *B*. *meridionalis* gut contents.

**Fig 5:**
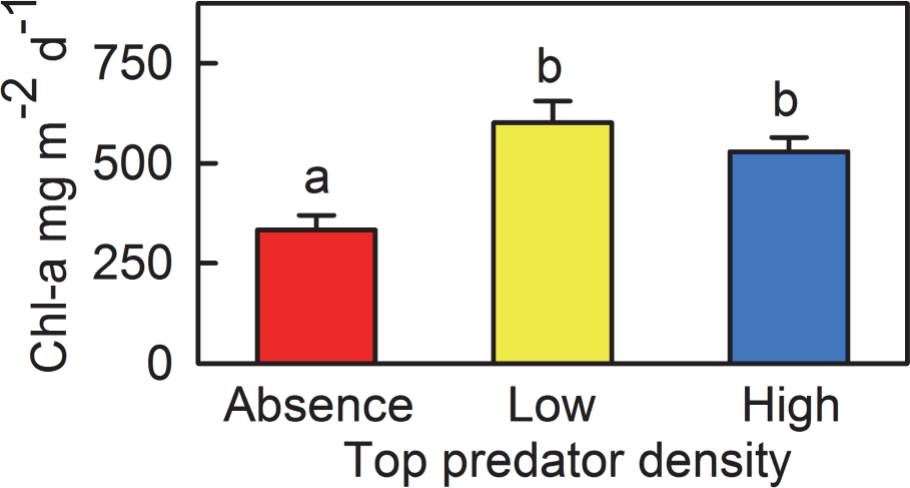
Periphyton net primary production measured as the chlorophyll-*a* on tiles for the three experimental treatments. Bars represent mean ± SE (mg m^-2^ d^-1^). Red bars = treatment without barbels; yellow bars = treatment with a low density of barbels; blue bars = treatment with a high density of barbels. Different letters correspond to significant differences resulting from the pairwise comparisons among treatments (*U*-test, p<0.05).

Periphyton primary production declined in the absence of *B*. *meridionalis* (χ^2^ = 17.82, p<0.001; Fig. 5, S1 Table).

## Discussion

This study demonstrated that apex consumer extinctions in intermittent streams may result in major changes to the system’s structure and function. Like others [8–10,54], our study showed how a top predator extirpation led to ‘mesopredator release’ in terms of abundance and richness. More importantly, top predator loss led to ‘prey release’, which contrasts with traditional food web theory and IGP literature. In addition, it triggered a trophic cascade that reduced periphyton primary production. Macroinvertebrate community composition also changed due to *B*. *meridionalis* absence. These results, along with other studies done in temporary salt marshes [55] and streams [56,57], support that the effects of the loss of small-bodied fish are equivalent to local extinctions of larger apex consumers in other ecosystems (e.g., the arctic fox, wolf, jaguar, sea otter or large reef fish [4,54,58]). Most studies about the consequences of the extinctions of top predators have been focused on large-bodied predators in terrestrial an marine systems [8,9], usually associating large-bodied species to the top of the food webs and small-bodied species to lower trophic levels [5,18], positing also that large-bodied species are at much greater extinction risk than smaller species (see [59]). However, as showed in this study, small-bodied fish in aquatic ecosystems may also exert strong top-down effects, supporting further that ‘keystone species’ is not a body-size dependent concept, ‘keystone species’ are those whose effects in the ecosystem are disproportionate to their abundance [60,61]. Moreover, Jenkins [62] suggests that aquatic species, in particular freshwater fish, are more vulnerable to extinction than terrestrial species, and Olden *et al*. [63] highlight that the most globally threatened freshwater fish are small-bodied species. Putting together the results of this study with the fact that numerous small-bodied freshwater fish are at extinction risk, it seems critical to persist in the consideration of the ecological consequences of their possible losses.

### Ecosystem structure: ‘mesopredator release’ and ‘prey release’

Mesopredators were more abundant in mesocosms lacking barbels, supporting the ‘mesopredator release’ hypothesis (see Fig. 1B), which confirms that the loss of small-bodied top predators may have this main common effect with large-bodied predator extirpations [8–10,54]. Several predatory invertebrates that characterised the enclosures lacking barbels (e.g. *Zavrelimyia* sp., *Parasigara* sp. and *Stictonectes* sp.; see Table 1) dominated barbel gut contents, indicating that fish predation contributed to density reduction for these taxa in the presence of barbels. Other taxa, such as *Chaoborus* sp., were not found in barbel gut contents, suggesting that the density decline for some taxa was likely the result of induced emigration. Mesopredator abundance thus appears to be controlled by the top predator through the combination of predation and possible non-consumption impacts such as competition or induced emigration. Moreover, mesopredator richness also increased in top predator absence. Consequently, a basic element of trophic webs was altered [64]: predator:prey ratios differed among the barbel density treatments (see Fig. 4E-F). Even though predator:prey richness ratio has been previously considered invariant due to underlying community assembly rules [65–67], our results support other studies that did not find conservative predator:prey ratios [68,69] and suggest that secondary and primary consumers respond unequally to the presence of a top predator.

‘Mesopredator release’ did not lead to a negative or a null effect on primary consumers (see Fig. 4A), which conflicts with the original IGP theory [13–15,70]. In contrast, top predator absence led to increased primary consumer abundance (i.e., ‘prey release’), which indicates that the top predator was more effective than mesopredators at suppressing prey. A growing body of literature has posited that top predator presence does not necessarily lead to higher prey abundance if the mesopredator exclusively uses alternate prey [71] or is cannibalistic [72]. However, these new perspectives on IGP are difficult to apply in empirical studies because models continue to oversimplify real food webs (e.g. by modelling food webs with just one intermediate predator). The IGP meta-analysis of Vance-Chalcraft *et al*. [73] concluded that top predator presence usually leads to ‘prey release’, as predicted by trophic cascade theory, however, it suggested that this is unclear in lotic ecosystems. In this sense, our results showed that the role of the apex consumer was not functionally replaced by the remaining species [74,75], suggesting that the predator assemblage is more important than diversity *per se* [6,76], with species identity being the critical factor.

Our study confirmed top predator extirpation modified the whole community composition. This finding was previously reported for intermittent streams exclusively by Williams *et al*. [31], who found fish have a top-down effect on macroinvertebrate assemblages in isolated pools. But to our knowledge, our study is the first in demonstrating top predator extirpation can change community composition in a running intermittent stream. The treatment lacking barbels was the only that contained a large number of associated indicator taxa (see Table 1). Therefore, the presence of *B*. *meridionalis* prompted a macroinvertebrate community that was a subset of the macroinvertebrate community without the top predator. The responses of invertebrate populations to barbel presence were highly taxon-dependent, which supports evidence elsewhere that taxa within a trophic level are not functionally equivalent [75,77]. No taxon was however positively affected by barbel presence. We found a statistically significant response even from highly mobile taxa that could rapidly recolonise the enclosures by drift [56,78], indicating a strong top-predator impact. These results indicate that some invertebrates have difficulty co-occurring with this apex consumer. Thus, the local extinction of *B*. *meridionalis* offered a competitive advantage for these vulnerable species to predation, and did not lead to an extinction cascade, which conflicts with the predator-mediated coexistence theory [16]. Likewise, it contrasts with several studies that relate top predator extinctions to a decline in biodiversity [9,12]; we did not find a relationship between top predator loss and total taxa richness or Simpson’s diversity, only for mesopredator richness that increased in top predator absence.

Several studies have emphasised that top predators may be functionally extinct from an ecosystem before being extirpated [18,54,79]. Management efforts to maintain threatened top predators at persistent levels can be ecologically irrelevant if the top predator population does not reach a functionally effective abundance. In our study, the top predator at low density (i.e., pre-fire density) led to an effective suppression of mesopredators, modified the whole macroinvertebrate community composition, and increased indirectly periphyton primary production, compared to the treatment without barbels. However, part of the top predator functional role was only revealed at higher fish density, since the suppression of mesopredator richness and primary consumers’ abundance did not occur at low top predator density. These results place apex consumer density as a continuum factor that modulates top predator effects in the ecosystem, confirming that studies about functional extinction thresholds that research top-down effects of apex consumers’ extinctions at different densities are particularly relevant for ecosystem restoration and conservation purposes.

### Ecosystem function: primary production response

Periphyton net primary production was significantly lower in the absence of *B*. *meridionalis* (see Fig. 5), confirming a strong trophic cascade effect that modified ecosystem function. This effect could occur through several different mechanisms, which are not necessarily mutually exclusive. Changes in primary consumer density could not fully explain the decline in primary production in top predator absence (see Fig. 4A). However, primary production could be top-down controlled by one or more taxa due to differences in the strength of this interaction, with herbivore identity being the key in the herbivore-producer interface. In this case, *B*. *meridionalis* extirpation could have increased the abundance of taxa that placed strong pressures on periphyton, triggering a trophic cascade without increasing the total abundance of primary consumers. Another explanation could be that predatory invertebrates were actually omnivorous, and ‘mesopredator release’ (see Fig. 4C) led to the increased consumption of periphyton. In addition to density-dependent causes, top predator presence could have led to higher primary production through a trait-mediated effect, reducing foraging activity by herbivores [77]. Although positive interactions have been studied less frequently by benthologists [2], *B*. *meridionalis* presence could have had a direct positive effect on periphyton production via nutrient release and/or by increasing light availability as a result of reduced sediment deposition through feeding foraging movements [35]. These results demonstrate that trophic cascades can be strengthened at the herbivore-producer interface, and contrast with those of Shurin *et al*. [80], which established that predators more strongly affected primary consumers compared to producers.

Our primary production results have implications for the management of natural and human-altered ecosystems. For instance, our results could modify the general view of how predatory fish abundance is linked to primary production in freshwater ecosystems, given that our results conflicted with traditional trophic cascade theory (which holds that each trophic level is related to the level above and below it in a direct and negative way [11]). In agroecosystems, biological-control practitioners often consider IGP, a very common interaction among aphidophagous predators and parasitoids [14,81]. In this context, Finke and Denno [15] advised against promoting diverse predator assemblages in which IGP was common because it would weaken the suppression of herbivore pests and reduce productivity. These kinds of generalisations can lead to ineffective management practices, particularly given that our results showed that IGP did not dampen the trophic cascade and that neither IGP nor diversity were linked to cascade strength. Instead, in agreement with Borer *et al*. [82], cascade strength depended on the identity of predators and herbivores. Therefore, we recommend that managers place more importance on species identity in decision-making processes to better predict management outcomes.

## Conclusions and Implications

We conclude that intermittent streams may be affected by the consequences of top predator extinctions. In this study, the apex consumer was functionally irreplaceable, despite its small-bodied size and even at low population densities, its local extinction led to the loss of an important functional role that resulted in major changes to the ecosystem. Top predator absence triggered a ‘mesopredator release’, but also a ‘prey release’, and changed the whole macroinvertebrate community composition. Regarding ecosystem function, periphyton primary production declined indirectly due to top predator loss. We highlighted that the consequences of this species loss were unforeseen, particularly given that our results were not supported by traditional food web theory. Which ecological responses in mesocosms can be extrapolated to real ecosystems is an open ecological question [83]. Brown et al. [84] demonstrated that aquatic mesocosms can reproduce replicable and realistically not just physicochemistry and macroinvertebrate community composition but complex food webs. Our in-stream mesocosms were carefully design to not be a methodological artefact: mesh size allowed macroinvertebrate emigration/inmigration, and complex tray subsrates within the mesocosms provided refuge to macroinvertebrates. However, spatial complexity and refuge diversity were probably lower in the mesocosms compared to natural stream conditions, which may have increased predator-prey encounter rates. On the other hand, we used conservative top predator densities (i.e., the stream’s pre-fire average density and its double); however, *B*. *meridionalis* can reach higher densities in stream isolated pools during the dry period (up to 20 ind m^-2^, usually in summer) suggesting that the impact of this top predator could be even higher than observed here. Thus, despite of the limitations of our study, our main result is consistent, the extirpation of a small-bodied top predator can led to deep system changes in an intermittent stream, at least in the hydrological conditions during our experiment. However, research at larger spatial and temporal scales is needed to integrate the impact of hydrological variability in intermittent streams.

Small-bodied freshwater fish species usually lack commercial value and are often overlooked in conservation management even when considered threatened [85,86]. Based on our results, we recommend that reintroduction programs be considered for small-bodied fish in intermittent streams, where species such *B*. *meridionalis* had become extirpated. Reintroduction programs would allow not just for recovery of endangered species populations (e.g., *B*. *meridionalis*), but for the restoration of the ecosystem. Likewise, reintroductions should be considered within a restoration ecology framework, not focusing on mere species presence, but on ecological effectiveness. Because habitat fragmentation often drives apex consumer extirpations [10,87] and can hinder following natural recolonisation, we also recommend the improvement of ecosystem connectivity as a preventive tool as well as a first step in restoration programs. In the context of freshwater ecosystems’ conservation, given the high extinction risk of small-bodied freshwater fish, our study evidences that unpredictable ecosystem changes in these ecosystems may occur if conservation efforts are not undertaken.

## Supporting Information

S1 Table Taxa abundance and periphyton primary production data for the three experimental treatments. Taxa are sorted by decreasing abundance in the treatment without top barbels. Category: 1 = primary consumer; 2 = secondary consumer.
(DOCX)
