# Supplementary material for: Small but Powerful: Top Predator Local Extinction Affects Ecosystem Structure and Function in an Intermittent Stream
Source: PLoS One. 2015 Feb 25;10(2):e0117630. doi: 10.1371/journal.pone.0117630 (PMC4340793; doi:10.1371/journal.pone.0117630)
Supplement: S1 Table — Taxa are sorted by decreasing abundance in the treatment without top barbels. Category: 1 = primary consumer; 2 = secondary consumer. (DOCX) [file pone.0117630.s001.docx]

**Supporting Information**

**Table S1. Taxa abundance and periphyton primary production data for the three experimental treatments.**

Taxa are sorted by decreasing abundance in the treatment without barbels. Category: 1 = primary consumer; 2 = secondary consumer.

|  | **Category** | **Without top predator** | | **Low top predator density** | | **High top predator density** | |
| --- | --- | --- | --- | --- | --- | --- | --- |
| **Taxa (ind m^-2^)** |  | mean | SE | mean | SE | mean | SE |
| *Tanytarsus* sp. | 1 | 4882.5 | 890.2 | 3334.3 | 817.0 | 1955.8 | 543.8 |
| *Zavrelimyia* sp. | 2 | 2433.7 | 330.7 | 1928.2 | 153.3 | 1132.3 | 135.9 |
| *Cricotopus* sp. | 1 | 2003.3 | 475.4 | 1468.0 | 243.2 | 831.2 | 132.2 |
| *Procladius* sp. | 2 | 1203.6 | 352.1 | 283.9 | 108.5 | 361.3 | 172.1 |
| *Habrophlebia* sp. | 1 | 1114.3 | 209.6 | 708.5 | 57.6 | 456.2 | 33.6 |
| Cladocera | 1 | 1034.7 | 247.5 | 557.8 | 97.2 | 256.3 | 44.4 |
| *Dicrotendipes* sp. | 1 | 888.1 | 214.6 | 1644.4 | 372.5 | 948.9 | 162.0 |
| *Corynoneura* sp. | 1 | 673.6 | 148.6 | 932.4 | 123.9 | 634.2 | 100.1 |
| *Gyraulus* sp. | 1 | 651.8 | 140.4 | 667.2 | 100.9 | 519.5 | 114.4 |
| *Paratanytarsus* sp. | 1 | 560.7 | 255.9 | 878.1 | 198.2 | 506.5 | 186.4 |
| *Microtendipes* sp. | 1 | 546.3 | 88.0 | 714.4 | 284.9 | 1329.5 | 988.3 |
| *Radix* sp. | 1 | 449.6 | 92.6 | 257.1 | 74.0 | 136.3 | 31.4 |
| Ostracoda | 1 | 396.7 | 67.9 | 146.1 | 34.7 | 159.0 | 40.5 |
| *Hydra* sp. | 2 | 373.2 | 87.9 | 375.1 | 105.6 | 105.1 | 28.3 |
| *Nanocladius* sp. | 1 | 321.9 | 63.5 | 437.0 | 119.1 | 232.6 | 38.5 |
| *Helobdella stagnalis* | 2 | 279.5 | 47.9 | 175.8 | 48.1 | 201.9 | 43.5 |
| Oligochaeta | 1 | 275.9 | 93.8 | 238.3 | 47.1 | 81.9 | 17.7 |
| *Phaenopsectra* sp. | 1 | 221.4 | 106.8 | 216.9 | 87.3 | 98.8 | 39.5 |
| *Chironomus* sp. | 1 | 188.9 | 95.5 | 131.2 | 65.2 | 36.3 | 27.6 |
| *Chalcolestes viridis* | 2 | 175.5 | 24.2 | 59.0 | 5.1 | 39.6 | 4.4 |
| *Baetis* sp. | 1 | 148.6 | 54.1 | 146.5 | 15.1 | 34.6 | 16.2 |
| *Stictonectes* sp. | 2 | 132.9 | 26.2 | 46.5 | 11.4 | 32.6 | 4.4 |
| *Cloeon* sp. | 1 | 116.5 | 29.0 | 17.4 | 5.7 | 30.5 | 7.6 |
| *Acentrella* sp. | 1 | 109.5 | 38.2 | 126.5 | 26.0 | 64.5 | 21.8 |
| *Caenis* sp. | 1 | 94.9 | 21.7 | 75.5 | 9.7 | 64.8 | 15.2 |
| *Parasigara* sp. | 2 | 81.3 | 16.9 | 25.8 | 5.8 | 10.7 | 3.3 |
| *Rheotanytarsus* sp. | 1 | 71.9 | 71.9 | 371.9 | 306.9 | 43.7 | 25.3 |
| *Cladotanytarsus* sp. | 1 | 70.8 | 70.8 | 0.0 | 0.0 | 0.0 | 0.0 |
| *Polypedilum* sp. | 1 | 62.4 | 26.4 | 48.3 | 42.7 | 126.5 | 77.4 |
| *Chaoborus* sp. | 2 | 61.1 | 24.4 | 4.2 | 1.8 | 0.7 | 0.7 |
| Copepoda | 1 | 40.1 | 16.4 | 77.3 | 15.5 | 20.1 | 12.2 |
| *Sympetrum* sp. | 2 | 37.5 | 6.9 | 33.3 | 7.5 | 13.9 | 3.3 |
| Dasyheleinae | 1 | 35.9 | 12.3 | 9.7 | 3.0 | 8.9 | 3.7 |
| *Pelophylax perezi* | 1 | 23.6 | 11.3 | 4.2 | 1.5 | 10.4 | 4.9 |
| Hidracarina | 2 | 20.8 | 11.7 | 27.0 | 24.8 | 13.9 | 9.4 |
| *Haliplus* sp. | 1 | 17.4 | 16.6 | 0.0 | 0.0 | 4.2 | 2.1 |
| *Cyrnus* sp. | 2 | 16.8 | 8.8 | 10.5 | 4.4 | 7.6 | 2.7 |
| *Agabus* sp. | 2 | 15.3 | 6.7 | 2.1 | 1.0 | 1.4 | 0.9 |
| Terrestrial invertebrates | - | 13.2 | 12.4 | 2.8 | 1.5 | 2.1 | 1.5 |
| *Dugesia* sp. | 2 | 12.4 | 11.0 | 2.1 | 1.5 | 6.9 | 5.6 |
| *Physella* sp. | 1 | 9.7 | 6.4 | 7.6 | 2.3 | 2.8 | 1.5 |

|  | **Category** | **Without top predator** | | **Low top predator density** | | **High top predator density** | |
| --- | --- | --- | --- | --- | --- | --- | --- |
| **Taxa (ind m^-2^)** |  | mean | SE | mean | SE | mean | SE |
| *Aeshna* sp. | 2 | 9.0 | 3.9 | 4.9 | 2.3 | 1.4 | 0.9 |
| *Dixa* sp. | 1 | 8.7 | 5.4 | 2.8 | 1.6 | 5.5 | 2.6 |
| Coenagrionidae | 2 | 7.6 | 5.5 | 0.7 | 0.7 | 1.4 | 0.9 |
| *Notonecta* sp. | 2 | 7.6 | 5.4 | 4.1 | 1.8 | 1.4 | 0.9 |
| *Yola bicarinata* | 1 | 6.8 | 5.5 | 10.5 | 2.8 | 6.2 | 2.8 |
| *Oulimnius* sp. | 2 | 4.9 | 2.9 | 14.5 | 3.6 | 3.4 | 1.8 |
| *Ancylus fluviatilis* | 1 | 3.6 | 1.8 | 0.7 | 0.7 | 4.1 | 2.2 |
| *Deronectes* sp. | 2 | 2.8 | 2.1 | 0.0 | 0.0 | 0.0 | 0.0 |
| *Elmis* sp. | 1 | 2.8 | 2.8 | 0.0 | 0.0 | 0.0 | 0.0 |
| *Brachytron pratense* | 2 | 2.1 | 1.0 | 0.0 | 0.0 | 0.0 | 0.0 |
| *Gyrinus* sp. | 2 | 2.1 | 1.0 | 3.5 | 1.1 | 0.7 | 0.7 |
| *Nebrioporus* sp. | 2 | 2.1 | 1.5 | 2.8 | 1.5 | 2.1 | 1.1 |
| *Pisidium* sp. | 1 | 1.9 | 1.4 | 5.6 | 3.8 | 17.4 | 11.7 |
| *Psectrocladius* sp. | 1 | 1.4 | 1.0 | 18.1 | 18.1 | 42.4 | 28.8 |
| *Anax imperator* | 2 | 1.4 | 0.9 | 1.4 | 0.9 | 0.7 | 0.7 |
| Simuliidae | 1 | 1.4 | 1.4 | 1.4 | 0.9 | 4.4 | 2.8 |
| Hemerodromiinae | 2 | 0.7 | 0.7 | 3.5 | 2.1 | 0.0 | 0.0 |
| *Hydrometra stagnorum* | 2 | 0.7 | 0.7 | 2.3 | 2.3 | 0.0 | 0.0 |
| *Microvelia* sp. | 2 | 0.7 | 0.7 | 0.0 | 0.0 | 0.7 | 0.7 |
| *Esolus* sp. | 1 | 0.7 | 0.7 | 0.0 | 0.0 | 1.4 | 1.4 |
| *Ilybius* sp. | 2 | 0.7 | 0.7 | 0.0 | 0.0 | 0.0 | 0.0 |
| *Limnephilus* sp. | 1 | 0.7 | 0.7 | 0.0 | 0.0 | 0.7 | 0.7 |
| Pyralydae | 1 | 0.7 | 0.7 | 0.7 | 0.7 | 0.0 | 0.0 |
| *Serratella* sp. | 1 | 0.7 | 0.7 | 0.0 | 0.0 | 0.7 | 0.7 |
| *Sialis* sp. | 2 | 0.7 | 0.7 | 0.0 | 0.0 | 0.0 | 0.0 |
| *Tinodes* sp. | 1 | 0.7 | 0.7 | 0.7 | 0.7 | 3.5 | 2.8 |
| Stratiomyidae | 1 | 0.7 | 0.7 | 0.0 | 0.0 | 0.7 | 0.7 |
| *Atherix* sp. | 2 | 0.0 | 0.0 | 0.7 | 0.7 | 0.0 | 0.0 |
| *Brillia* sp. | 1 | 0.0 | 0.0 | 0.0 | 0.0 | 8.5 | 8.5 |
| *Limnophyes* sp. | 1 | 0.0 | 0.0 | 40.0 | 27.3 | 24.1 | 24.1 |
| *Mesovelia vittigera* | 2 | 0.0 | 0.0 | 0.6 | 0.6 | 0.0 | 0.0 |
| *Micropsectra* sp. | 1 | 0.0 | 0.0 | 0.0 | 0.0 | 30.6 | 30.6 |
| Muscidae | 2 | 0.0 | 0.0 | 0.0 | 0.0 | 0.7 | 0.7 |
| *Normandia* sp. | 1 | 0.0 | 0.0 | 0.7 | 0.7 | 0.0 | 0.0 |
| *Parametriocnemus* sp. | 1 | 0.0 | 0.0 | 0.0 | 0.0 | 17.1 | 17.1 |
| *Rheocricotopus* sp. | 1 | 0.0 | 0.0 | 12.0 | 12.0 | 17.4 | 17.4 |
| *Stictotarsus* sp. | 2 | 0.0 | 0.0 | 0.0 | 0.0 | 0.7 | 0.7 |
|  |  |  |  |  |  |  |  |
| **Primary production** |  | mean | SE | mean | SE | mean | SE |
| Chl-a mg m^-2^ d^-1^ | - | 332.9 | 37.5 | 601.5 | 53.9 | 528.9 | 35.8 |
